# FBXO2 promotes hepatocellular carcinoma progression and sorafenib resistance by targeting USP49 for proteasomal degradation

**DOI:** 10.3389/fimmu.2025.1660034

**Published:** 2025-09-16

**Authors:** Sirui Hang, Qingqing Wang, Jie Zhang, Yiwei Dong, Bile Hu, Peter Wang, Liu Xu

**Affiliations:** ^1^ Department of Surgery, Zhejiang Chinese Medical University., Hangzhou, Zhejiang, China; ^2^ Department of Hepatobiliary Pancreatic Surgery, The First Hospital of Jiaxing & The first affiliated hospital of Jiaxing University, Jiaxing, Zhejiang, China; ^3^ Department of Medicine, Beijing Zhongwei Medical Research Center, Beijing, China; ^4^ Department of Mechanical Engineering, Northeastern University, Boston, MA, United States

**Keywords:** Fbxo2, USP49, proliferation, sorafenib, resistance

## Abstract

**Introduction:**

Hepatocellular carcinoma (HCC) is a highly prevalent and lethal malignancy with limited treatment efficacy due to tumor heterogeneity and the development of drug resistance. Identifying novel molecular mechanisms that drive HCC progression and therapeutic resistance is critical. F-box only protein 2 (FBXO2), an E3 ubiquitin ligase, has recently been implicated in tumorigenesis. However, its role in HCC remains unclear.

**Methods:**

We employed CCK-8, EdU, Transwell, and wound healing assays to evaluate the functional role of FBXO2 in HCC cells. Furthermore, Western blotting, immunoprecipitation, *in vivo* ubiquitination assays, and cycloheximide chase analysis were conducted to investigate the molecular mechanisms through which FBXO2 contributes to tumor progression in HCC.

**Results:**

FBXO2 is significantly upregulated in HCC tissues and correlates with poor patient prognosis. Functional assays demonstrated that FBXO2 promotes HCC cell proliferation, migration, and invasion *in vitro*, while its silencing exerts tumor-suppressive effects. Mechanistically, FBXO2 directly binds to and targets the USP49 for ubiquitin-mediated proteasomal degradation. This degradation decreases USP49 stability and function, thereby enhancing oncogenic potential. Importantly, silencing USP49 reversed the inhibitory effects of FBXO2 knockdown, confirming the FBXO2/USP49 axis as a functional regulator of HCC aggressiveness. Furthermore, FBXO2 depletion significantly enhanced the sensitivity of HCC cells and xenograft tumors to sorafenib treatment.

**Conclusion:**

Collectively, our findings establish FBXO2 as a critical modulator of HCC progression and therapeutic resistance via USP49 degradation, highlighting FBXO2 as a promising therapeutic target for overcoming sorafenib resistance in HCC.

## 1 Introduction

Hepatocellular carcinoma (HCC), which accounts for approximately 75 – 85% of primary liver cancer cases, is the third leading cause of cancer-related death and the sixth most commonly diagnosed malignancy worldwide (1–3). Sorafenib was the first approved first-line drug for advanced HCC by the US Food and Drug Administration. However, the effectiveness of sorafenib is severely limited by acquired drug resistance (4). Immunotherapy has emerged as a promising treatment strategy for HCC, aiming to enhance antitumor immune responses and improve patient outcomes (5). Atezolizumab combined with bevacizumab remains the standard of care for first-line treatment of advanced HCC (6). Despite the availability of various therapeutic options, including immune checkpoint inhibitors, sorafenib, and lenvatinib, the objective response rate remains suboptimal (7, 8). This is largely due to interindividual variability in drug sensitivity, adverse side effects, and the emergence of drug resistance (9, 10). A deeper understanding of the mechanisms underlying HCC progression and therapeutic resistance is therefore essential to identify novel and effective diagnostic and therapeutic targets.

Post-translational modification (PTM) has been suggested to control protein function, stability, localization, and interactions. Common types of PTMs include phosphorylation, ubiquitination, acetylation, methylation, and glycosylation (11). Ubiquitination is common in which one or more ubiquitin molecules are covalently attached to a target protein (12). This post-translational modification occurs through a three-step enzymatic cascade: activation, conjugation, and ligation (13, 14). which are catalyzed by ubiquitin-activating enzymes (E1s), ubiquitin-conjugating enzymes (E2s), and ubiquitin ligases (E3s), respectively (15). Among these, the SCF (Skp1–Cullin1–F-box) complex represents the largest subfamily of E3 ligases in mammals, comprising Skp1, Cullin1, Rbx1, and a variable F-box protein (FBP) that determines substrate specificity (16). FBPs play a critical role in the ubiquitination pathway and have been reported to target their substrates and control tumorigenesis (17–20).

F-Box only protein 2 (FBXO2), also known as FBG1 or Fbs1, is a cytoplasmic protein that functions as a substrate recognition component of the SCF (SKP1–Cullin–F-box) E3 ubiquitin ligase complex (21). FBXO2 has been shown to regulate neuronal protein homeostasis by targeting multiple glycoproteins for ubiquitin-mediated degradation, playing an important role in both normal and pathological neuronal function (22). Furthermore, FBXO2 modulates insulin signaling by promoting the degradation of insulin receptors through the ubiquitin–proteasome pathway (23). Emerging evidence indicates that FBXO2 was closely linked to tumorigenesis in ovarian cancer, glioma and oral squamous cell carcinoma (24–26). However, whether FBXO2 contributes to HCC remains largely unknown.

Ubiquitin-specific peptidase 49 (USP49), a member of the deubiquitinase family, is involved in diverse oncogenic processes (27). USP49 controls a number of cellular processes linked to tumor development by reversing ubiquitination, thereby modifying protein stability and cellular communication pathways (28). Notably, USP49 has garnered attention for its involvement in chemoresistance, wherein abnormal expression or dysregulated activity of USP49 contributes to the resistance of multiple cancer types to chemotherapeutic agents (29, 30). Given its role in drug resistance mechanisms, USP49 may also be implicated in the development of resistance to sorafenib in HCC (31). In the current study, we explored the functions of FBXO2 in HCC progression and sorafenib resistance. We reported that FBXO2 promoted cell proliferation, invasion, and sorafenib resistance via targeting USP49 for degradation in HCC.

## 2 Materials and methods

### 2.1 Cell culture

HCC cell lines, including HepG2 and Huh-7, were purchased from Beyotime Biotechnology Company (Shanghai, China). HepG2 cells are widely used in tumor biology research due to their stable proliferation but are non-permissive to hepatitis B virus (HBV) and hepatitis C virus (HCV) infection. Huh-7 cells are well-differentiated hepatocellular carcinoma cells and are permissive to HCV infection. Cells were cultured in Dulbecco’s Modified Eagle Medium (DMEM) containing 10% fetal bovine serum (FuHeng, Shanghai, China). Cells were maintained in a humidified incubator under 5% CO_2_ at 37°C.

### 2.2 CCK-8 cell viability assay

HCC cells were seeded into 96-well plates at a final density of 3.0 × 10^3^ cells per well. Cells were incubated for 0, 24, 48, 72, or 96 hours. At each time point, 10 μl of CCK-8 reagent (Dojindo, Japan) was added to each well, followed by a 2-hour incubation at 37°C in the dark. A microplate reader (TECAN, Switzerland) was used to measure the OD value at 450 nm (32).

### 2.3 EdU proliferation assay

Cell proliferation was evaluated using the BeyoClick™ EdU-594 Cell Proliferation Kit (Beyotime Biotechnology, China). HCC cells were seeded into 24-well plates at a density of 5.0 × 10^4^ cells per well. After 24 hours, cells were incubated with EdU solution for 4 hours, then fixed with 4% paraformaldehyde and permeabilized using the supplied permeabilization buffer. Cells were subsequently stained with Alexa Fluor 594, and nuclei were counterstained with Hoechst 33342. Fluorescence images were acquired using a fluorescence microscope, and the percentage of EdU-positive cells was quantified using ImageJ software (NIH, Rockville, USA).

### 2.4 Transwell assay

Cell invasion was assessed using a Transwell chamber assay. Briefly, Transwell inserts (Corning, USA) were pre-soaked at 37°C for 30 minutes and coated with Matrigel matrix diluted 1:8 in DMEM. For the invasion assay, 5 × 10^4^ cells suspended in 200 μL of serum-free DMEM were seeded into the upper chamber, while 500 μL of DMEM containing 20% fetal bovine serum was added to the lower chamber as a chemoattractant. After 24 hours of incubation at 37°C, non-invading cells on the upper surface were removed, and the invaded cells on the lower membrane were fixed and stained with 0.1% crystal violet. Images were acquired using an inverted microscope. For the migration assay, Transwell inserts were used without Matrigel coating. Migratory cells were stained with Calcein-AM (Beyotime, China) and visualized under a fluorescence microscope.

### 2.5 Wound healing assay

HCC cells were seeded in 6-well plates and allowed to grow to 90 – 100% confluence. A linear scratch was created using a sterile 200 μL pipette tip. The wells were washed gently with sterile PBS to remove debris and non-adherent cells. Wound closure was monitored by capturing images at 0 and 20 hours using an optical microscope. The migration rate was quantified by measuring the gap distance using ImageJ software.

### 2.6 Western blotting analysis

Total protein was extracted using RIPA lysis buffer and quantified by a BCA assay. Equal amounts of protein were separated via SDS-PAGE and transferred onto nitrocellulose membranes. After blocking with 5% non-fat milk for 1 hour at room temperature, membranes were incubated overnight at 4°C with primary antibodies, including anti-FBXO2 (14590-1-AP, Proteintech), anti-USP49 (18066-1-AP, Proteintech), anti-Myc (16286-1-AP, Proteintech), anti-GAPDH (GB15004-100, Servicebio), anti-Flag (F1804, Sigma), and anti-ubiquitin (sc-166553, Santa Cruz Biotechnology). The membranes were then washed and incubated with appropriate HRP-conjugated secondary antibodies. Signals were detected using Omni-ECL™ chemiluminescent substrate (YEASEN, Shanghai, China) (33).

### 2.7 Immunoprecipitation

Cell lysates were prepared from HepG2 or Huh-7 cells using NP-40 RIPA buffer. Co-immunoprecipitation was performed by incubating 5 μg of the indicated antibody with cell lysates overnight at 4°C, followed by the addition of protein A/G magnetic beads (B23202, Selleck). After incubation, the beads were washed thoroughly, and bound proteins were eluted by boiling in loading buffer. The eluates were then subjected to SDS-PAGE and Western blot analysis (34).

### 2.8 *In vivo* ubiquitination and CHX analysis

For *in vivo* ubiquitination assays, cells with or without plasmid transfection were treated with 20 μM MG-132 (a proteasome inhibitor) for 6 hours. Cells were then lysed, and ubiquitinated proteins were detected by immunoprecipitation followed by Western blotting using the appropriate antibodies. For CHX chase experiments, HepG2 and Huh-7 cells were treated with 100 μg/mL cycloheximide (CHX) to inhibit protein synthesis. Cells were harvested at 0, 2, 4, 6, and 8 hours post-treatment. Protein lysates were prepared using RIPA buffer and analyzed by SDS-PAGE followed by immunoblotting to evaluate protein stability over time.

### 2.9 *In vivo* nude mice xenograft studies

HepG2-shNC and HepG2-shFBXO2 cells (5 × 10^6^ in 100 μL PBS) were subcutaneously injected into BALB/c nude mice (n = 10). Once tumor volumes reached approximately 100 mm³, mice were randomly assigned into two groups and treated with either 20 mg/kg sorafenib or vehicle control via daily intraperitoneal injection for 20 days. Tumor volumes were measured every four days and calculated using the formula: V = (L × W²) × 0.52, where L is the longest diameter and W is the shortest diameter of the tumor. At the end of the treatment period, tumors were excised and weighed for further analysis.

### 2.10 Statistical analysis

All statistical analyses were performed using GraphPad Prism 8.0 or SPSS Statistics software. Data are presented as the mean ± standard deviation (SD). Comparisons between two groups were made using Student’s t-test, while one-way ANOVA was used for comparisons among multiple groups. A p-value < 0.05 was considered statistically significant.

## 3 Results

### 3.1 Elevated FBXO2 expression in HCC correlates with poor patient prognosis

**Figure 1:**
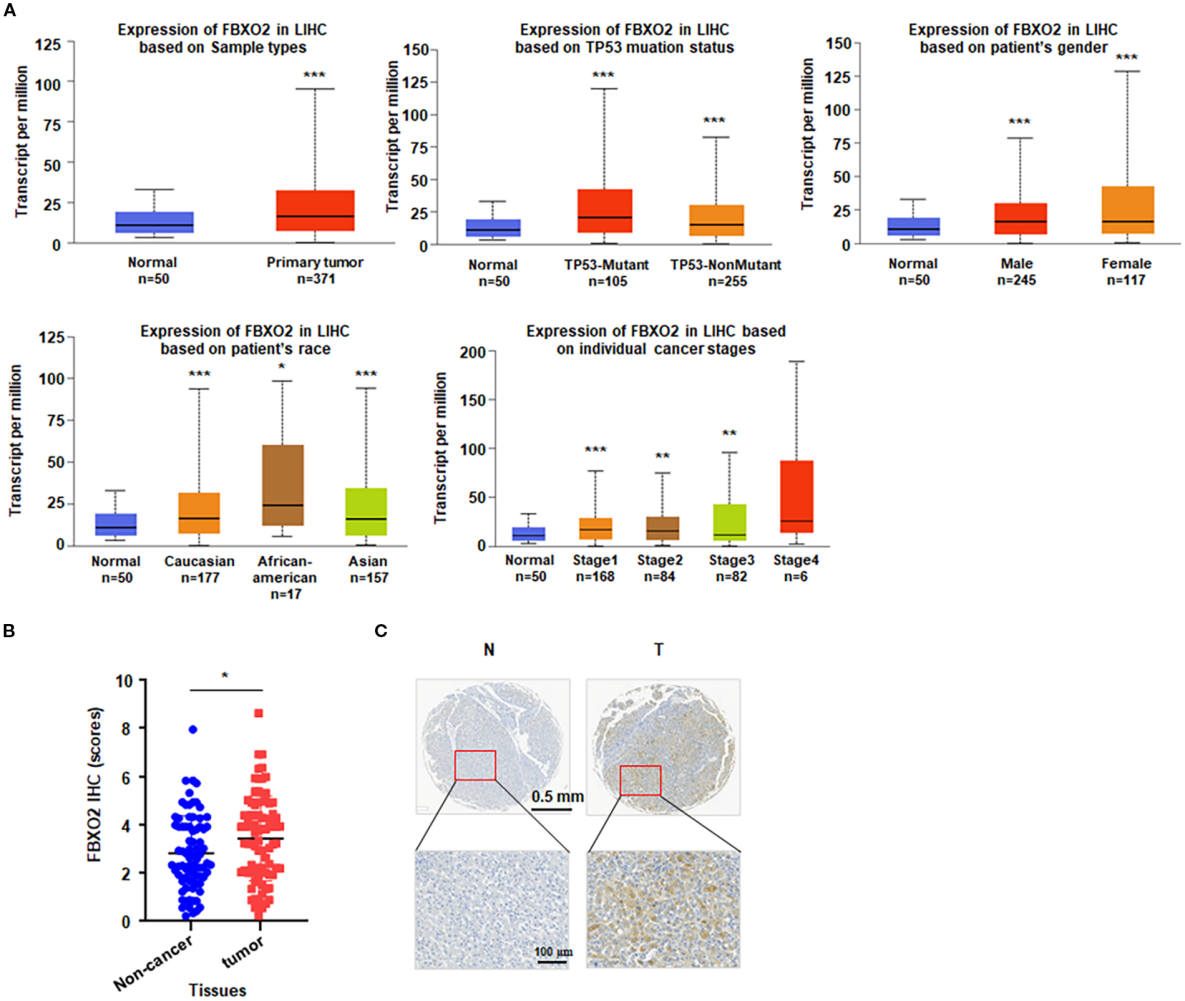
FBXO2 is upregulated in Liver hepatocellular carcinoma (LIHC). **(A)** FBXO2 expression in LIHC is stratified by TP53 mutation status, patient gender, race, and cancer stage using the UALCAN database. **(B)** Quantification of FBXO2 immunohistochemistry (IHC) scores in 80 LIHC samples and paired adjacent non-tumor tissues. **(C)** Representative IHC staining of FBXO2 in LIHC and matched normal tissues. N, normal tissues; T, tumor tissues. *p < 0.05, **p < 0.01, ***p < 0.001.

To investigate the expression pattern of FBXO2 in HCC, we analyzed publicly available transcriptomic data from The Cancer Genome Atlas (TCGA). The results revealed that FBXO2 expression was significantly upregulated in tumor tissues compared to adjacent normal liver tissues. To further explore the clinical relevance of FBXO2, we utilized the UALCAN database to assess its association with various clinicopathological features of HCC. Elevated FBXO2 expression was found to be significantly correlated with several clinical parameters, including TP53 mutation status, patient gender, race, and tumor stage (**Figure 1A**). In addition, immunohistochemistry (IHC) analysis confirmed that FBXO2 protein levels were markedly elevated in HCC tissues relative to adjacent normal liver tissues (**Figures 1B–C**). Furthermore, FBXO2 expression is associated with patient survival in HCC, as analyzed using the KMplot database (https://kmplot.com/analysis). These findings suggest that FBXO2 overexpression may play a role in HCC progression and could serve as a potential prognostic biomarker.

### 3.2 Depletion of FBXO2 inhibits proliferation, invasion, and migration of HCC cells

**Figure 2:**
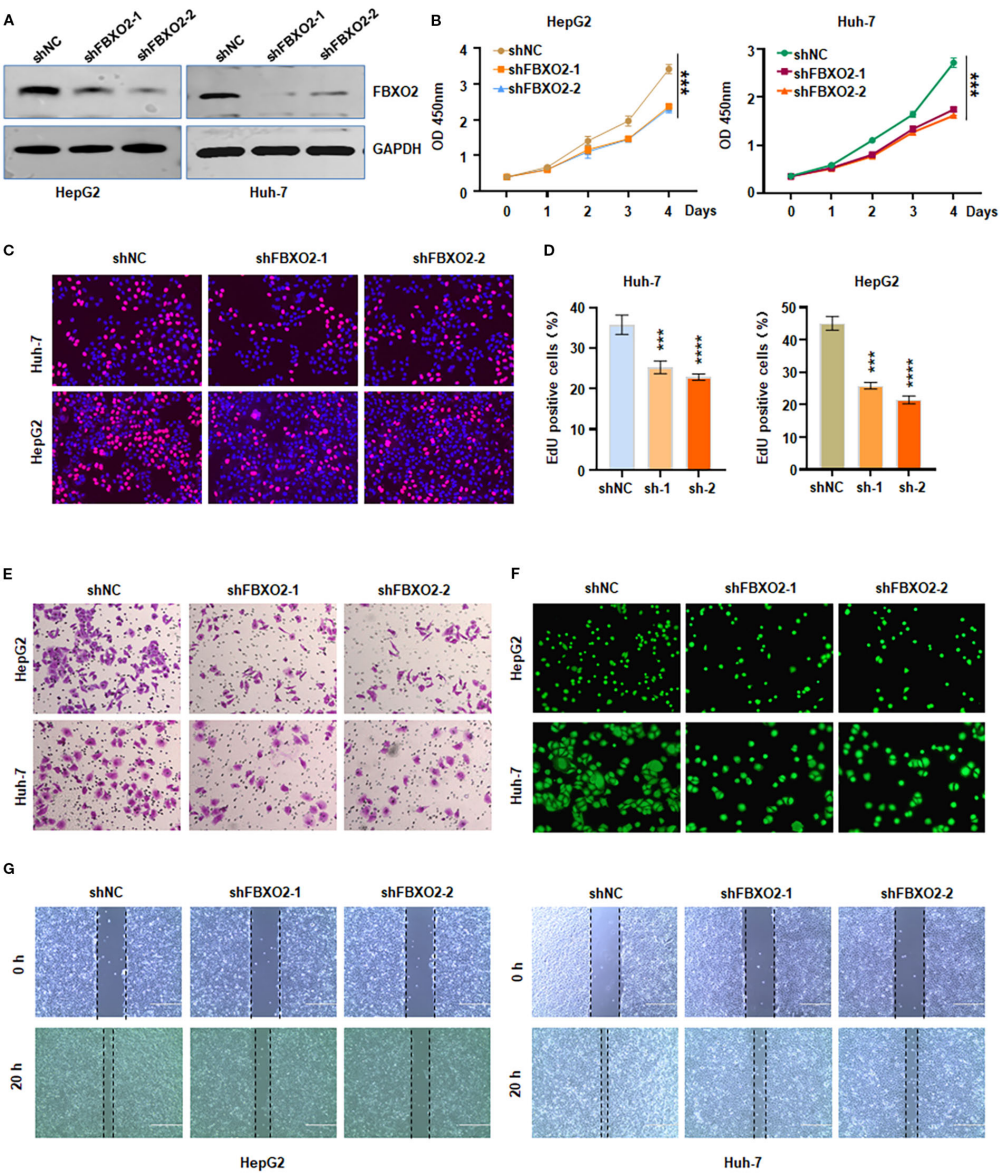
Knockdown of FBXO2 inhibits proliferation and motility of HCC cells. **(A)** Western blot (IB) analysis of whole cell lysates (WCLs) from HepG2 and Huh-7 cells infected with control shRNA (shNC) or FBXO2-specific shRNA (shFBXO2). **(B, C)** Cell proliferation assessed by CCK-8 and EdU assays following FBXO2 knockdown in HepG2 and Huh-7 cells. **(D)** Quantification of EdU-positive cells from **(C)**. shNC, shRNA negative control; sh-1, shFBXO2-1; sh-2, shFBXO2-2. **(E)** Cell invasion measured by Transwell assay in control and FBXO2-knockdown cells. **(F)** Cell migration analyzed using a calcein-AM-stained Transwell assay in control and FBXO2-knockdown cells. **(G)** Wound healing assay showing reduced migration in FBXO2-knockdown HepG2 and Huh-7 cells. **p < 0.01, ***p < 0.001.

To investigate the biological function of FBXO2 in HCC, we silenced its expression by transfecting HepG2 and Huh-7 cells with lentivirus encoding FBXO2-specific shRNA (**Figure 2A**). Knockdown of FBXO2 significantly reduced the proliferative capacity of HCC cells, as demonstrated by both CCK-8 and EdU assays (**Figures 2B–D**). To assess the impact on cell motility, we performed Transwell and wound healing assays. FBXO2 depletion markedly inhibited the invasion and migration of HepG2 and Huh-7 cells compared to control groups (**Figures 2E–G**). These results collectively indicate that FBXO2 knockdown significantly suppresses HCC cell proliferation and motility.

### 3.3 Overexpression of FBXO2 promotes cell proliferation, invasion, and migration of HCC cells

**Figure 3:**
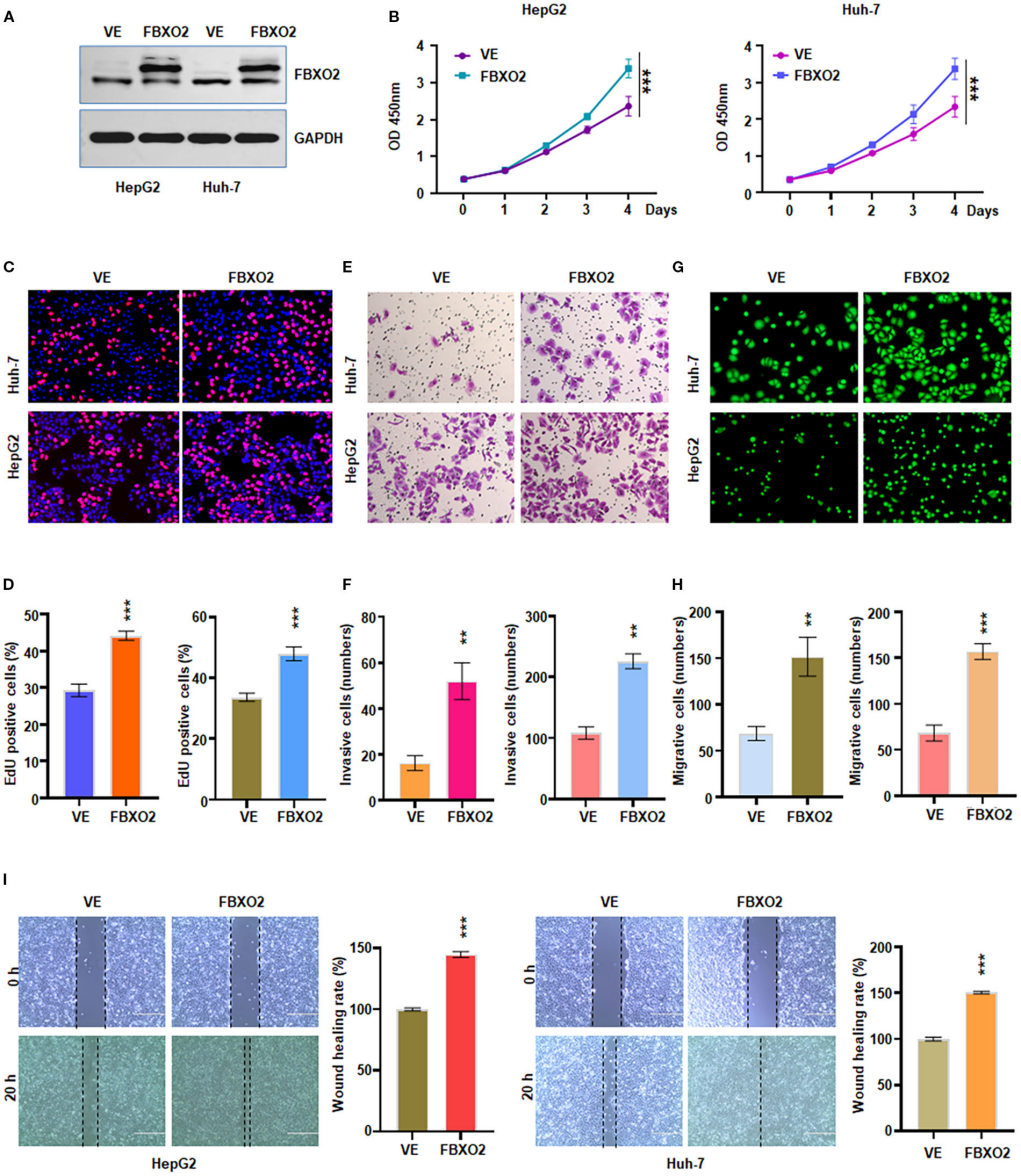
Overexpression of FBXO2 accelerated proliferation and motility of HCC cells. **(A)** Western blot showing FBXO2 protein levels in HepG2 and Huh-7 cells infected with Flag-FBXO2 or empty vector control lentivirus. VE, vector empty. **(B)** Cell proliferation assessed by CCK-8 assay in FBXO2-overexpressing cells. **(C)** EdU assay evaluating the effect of FBXO2 overexpression on cell proliferation. **(D)** Quantification of EdU-positive cells from **(C)**. **(E)** Transwell invasion assay demonstrating increased invasive ability of FBXO2-overexpressing cells. **(F)** Quantification of invaded cells from **(E)**. **(G)** Cell migration assessed by calcein-AM-stained Transwell assay in FBXO2-overexpressing cells. **(H)** Quantification of migrated cells from **(G)**. **(I)** Wound healing assay showing enhanced migration in FBXO2-overexpressing HepG2 and Huh-7 cells. **p < 0.01, ***p < 0.001.

To further validate the role of FBXO2, we generated FBXO2-overexpressing cell models by transfecting HepG2 and Huh-7 cells with a Flag-FBXO2 lentiviral construct (**Figure 3A**). CCK-8 and EdU assays revealed that FBXO2 overexpression significantly enhanced the proliferation of both cell lines (**Figures 3B–D**). In Transwell invasion assays, FBXO2-overexpressing HCC cells exhibited a significantly greater invasive ability compared to wild-type controls (**Figures 3E–F**). Furthermore, both Transwell and wound healing assays confirmed that FBXO2 overexpression significantly increased the migratory capacity of HCC cells (**Figures 3G–I**). Collectively, these results demonstrate that FBXO2 functions as a positive regulator of HCC cell proliferation, invasion, and migration.

### 3.4 FBXO2 binds to and degrades USP49

**Figure 4:**
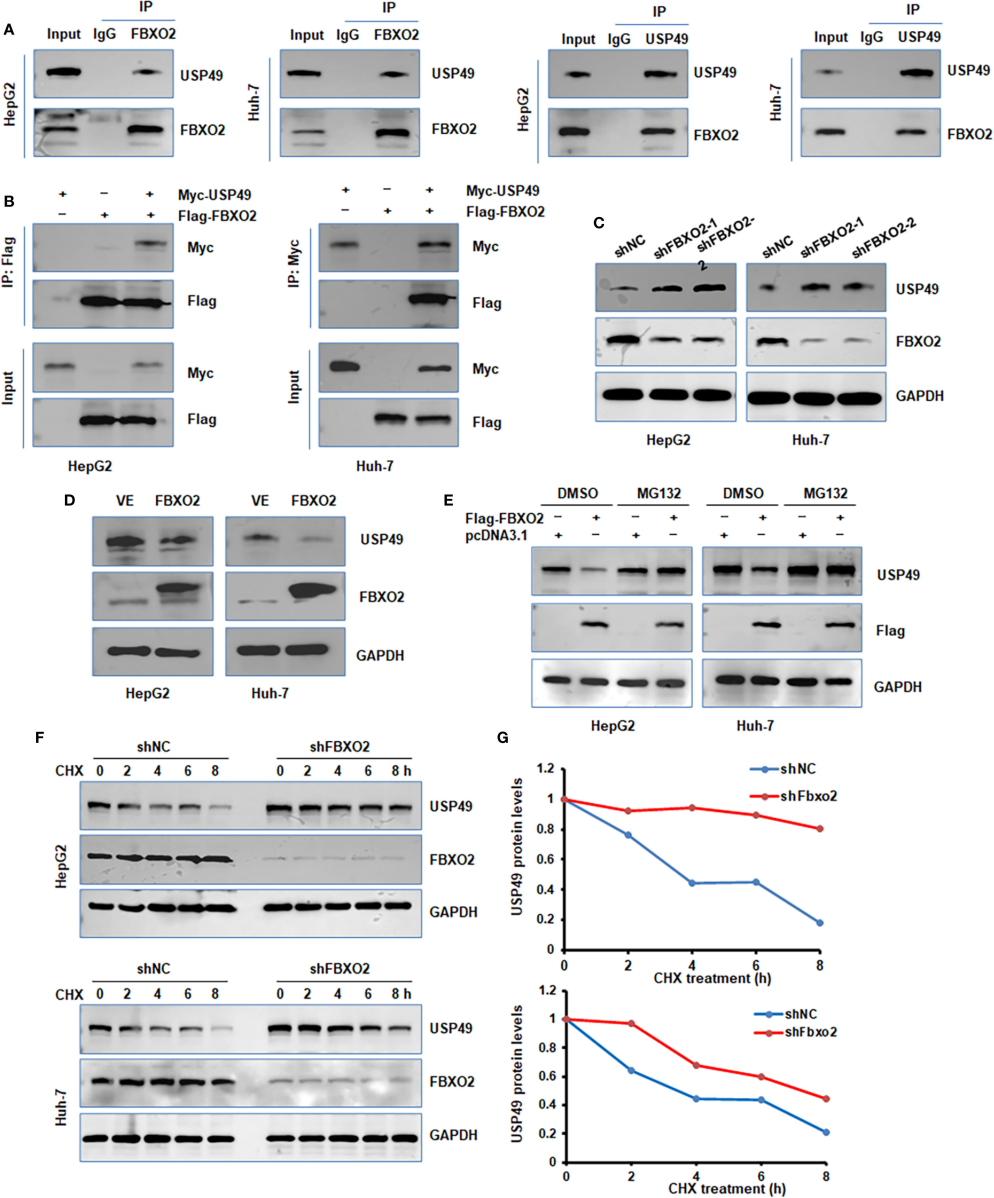
FBXO2 binds to and regulates protein stability of USP49. **(A)** Endogenous interaction between FBXO2 and USP49 was examined by immunoprecipitation in HepG2 and Huh-7 cells. **(B)** Co-immunoprecipitation was performed in HepG2 and Huh-7 cells transfected with Flag-FBXO2 and Myc-USP49 to detect exogenous interaction. **(C)** Western blot (WB) analysis of whole cell lysates (WCLs) from HepG2 and Huh-7 cells infected with FBXO2 shRNA or control shRNA lentivirus. **(D)** WB analysis of WCLs from HepG2 and Huh-7 cells infected with Flag-FBXO2 or empty vector lentivirus. **(E)** MG132 (proteasome inhibitor) treatment restored the reduction in USP49 protein levels caused by FBXO2 overexpression. **(F)** USP49 protein half-life was assessed by CHX-chase assay in HepG2 and Huh-7 cells with or without FBXO2 knockdown. **(G)** Quantification of USP49 protein levels over time from **(F)**, shown as a protein stability curve. shNC, shRNA negative control.

To identify potential substrates of Fbxo2, we performed liquid chromatography-tandem mass spectrometry (LC-MS/MS). Following transient transfection of Flag-tagged Fbxo2 into HEK293T cells, Fbxo2-associated protein complexes were immunoprecipitated and subjected to LC-MS/MS analysis, which revealed USP49-derived peptides as candidate substrates. To determine whether USP49 is a substrate of FBXO2, we performed co-immunoprecipitation (co-IP) assays in HepG2 and Huh-7 cells. The results confirmed both endogenous and exogenous interactions between FBXO2 and USP49 (**Figures 4A–B**). Given this interaction, we next examined how FBXO2 expression affects USP49 protein levels. Western blot analysis revealed that FBXO2 knockdown significantly increased USP49 protein levels, while FBXO2 overexpression led to a marked decrease (**Figures 4C, D**). Furthermore, the reduction in USP49 levels caused by FBXO2 overexpression was reversed by treatment with MG132, a proteasome inhibitor, indicating that FBXO2 mediates USP49 degradation through the ubiquitin-proteasome pathway (**Figure 4E**). To assess the effect of FBXO2 on USP49 protein stability, we performed a CHX chase assay. The results showed that FBXO2 depletion markedly delayed the degradation of USP49 (**Figures 4F–G**), further supporting the role of FBXO2 in promoting USP49 proteasomal degradation.

### 3.5 FBXO2 mediates USP49 ubiquitination via the FBA domain

**Figure 5:**
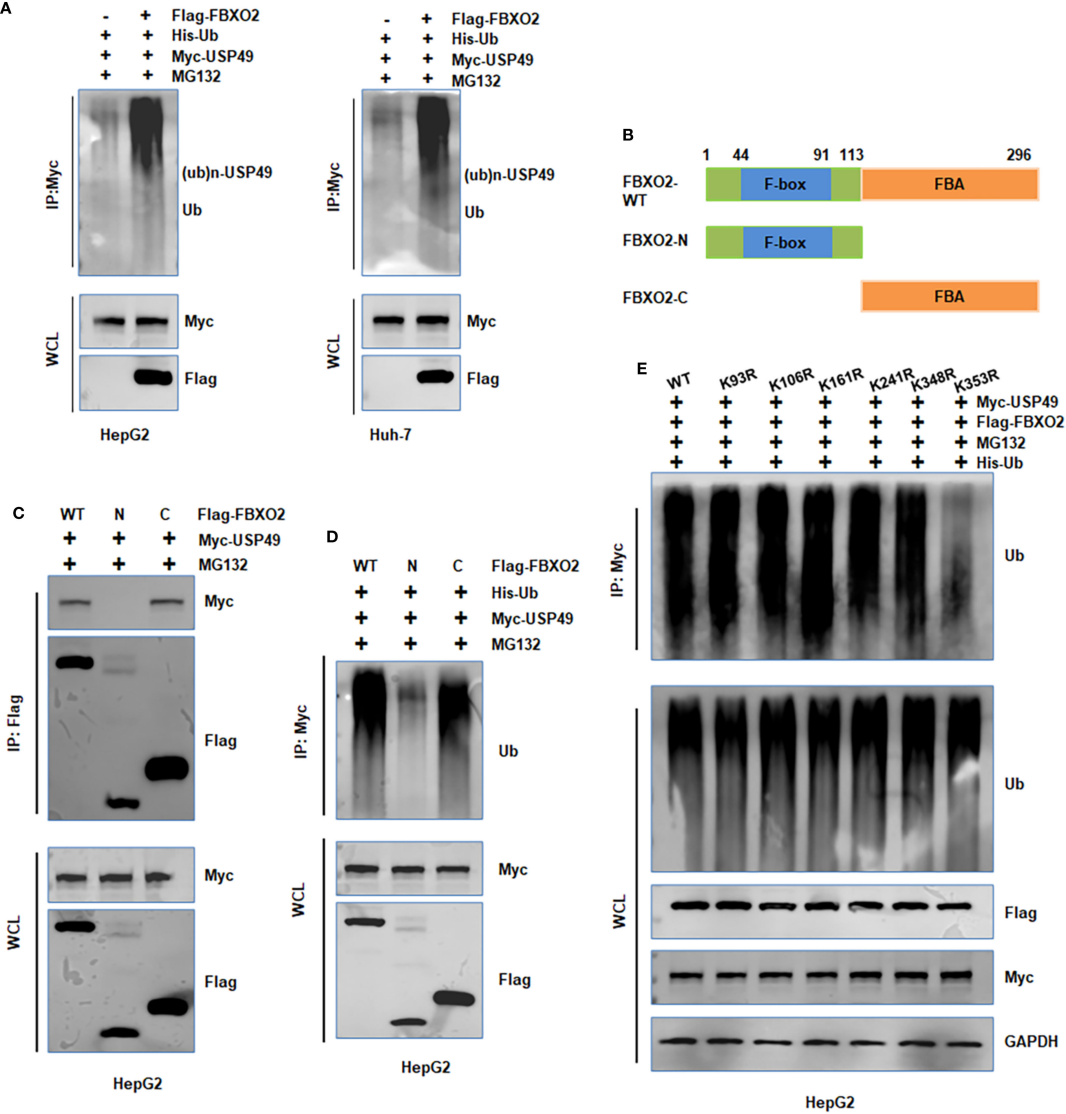
FBXO2 promotes the ubiquitination of USP49. **(A)** HepG2 and Huh-7 cells were transfected with Flag-FBXO2, His-Ub, and Myc-USP49 plasmids. After 12-hour treatment with MG132 (10 μM), cell lysates and Myc immunoprecipitates were analyzed by immunoblotting to detect USP49 ubiquitination. **(B)** Schematic representation of the FBXO2 protein domains and deletion mutants used in this study. **(C, D)** HepG2 cells were transfected with full-length or mutant FBXO2 constructs, along with Myc-USP49 and His-Ub. Cells were treated with MG132 (10 μM) for 12 hours. Whole cell lysates and immunoprecipitates were subjected to WB to assess USP49 ubiquitination. **(E)** HepG2 cells were co-transfected with Flag-FBXO2, His-Ub, and various Myc-tagged USP49 lysine mutants (K93R, K106R, K161R, K241R, K348R, K353R). Cell lysates were immunoprecipitated with Myc antibody and probed with anti-ubiquitin antibody to assess site-specific ubiquitination of USP49.

To further investigate the mechanism by which FBXO2 regulates USP49, we performed *in vivo* ubiquitination assays in HepG2 and Huh-7 cells. Overexpression of FBXO2 significantly enhanced the ubiquitination of USP49 (**Figure 5A**). To determine which domain of FBXO2 is responsible for USP49 recognition, we conducted co-IP experiments with FBXO2 deletion mutants. The results indicated that the F-box-associated (FBA) domain of FBXO2 is required for its interaction with USP49 (**Figures 5B–C**). Consistently, deletion of the FBA domain abolished FBXO2-mediated ubiquitination of USP49, as confirmed by *in vivo* ubiquitination assays (**Figure 5D**). Since ubiquitination typically occurs on lysine residues, we used the GPS-Uber prediction tool to identify potential ubiquitination sites on USP49. Six lysine-to-arginine mutants were constructed (K93R, K106R, K161R, K241R, K348R, and K353R). Among these, the K353R mutant exhibited markedly reduced ubiquitination compared to the wild-type and other mutants, suggesting that lysine 353 is a critical site for FBXO2-mediated ubiquitination (**Figure 5E**). Collectively, these results demonstrate that FBXO2 binds to USP49 via its FBA domain and promotes its degradation by enhancing ubiquitination at lysine 353 in HCC cells.

### 3.6 FBXO2 depletion reduces cell proliferation and motility via USP49 regulation

**Figure 6:**
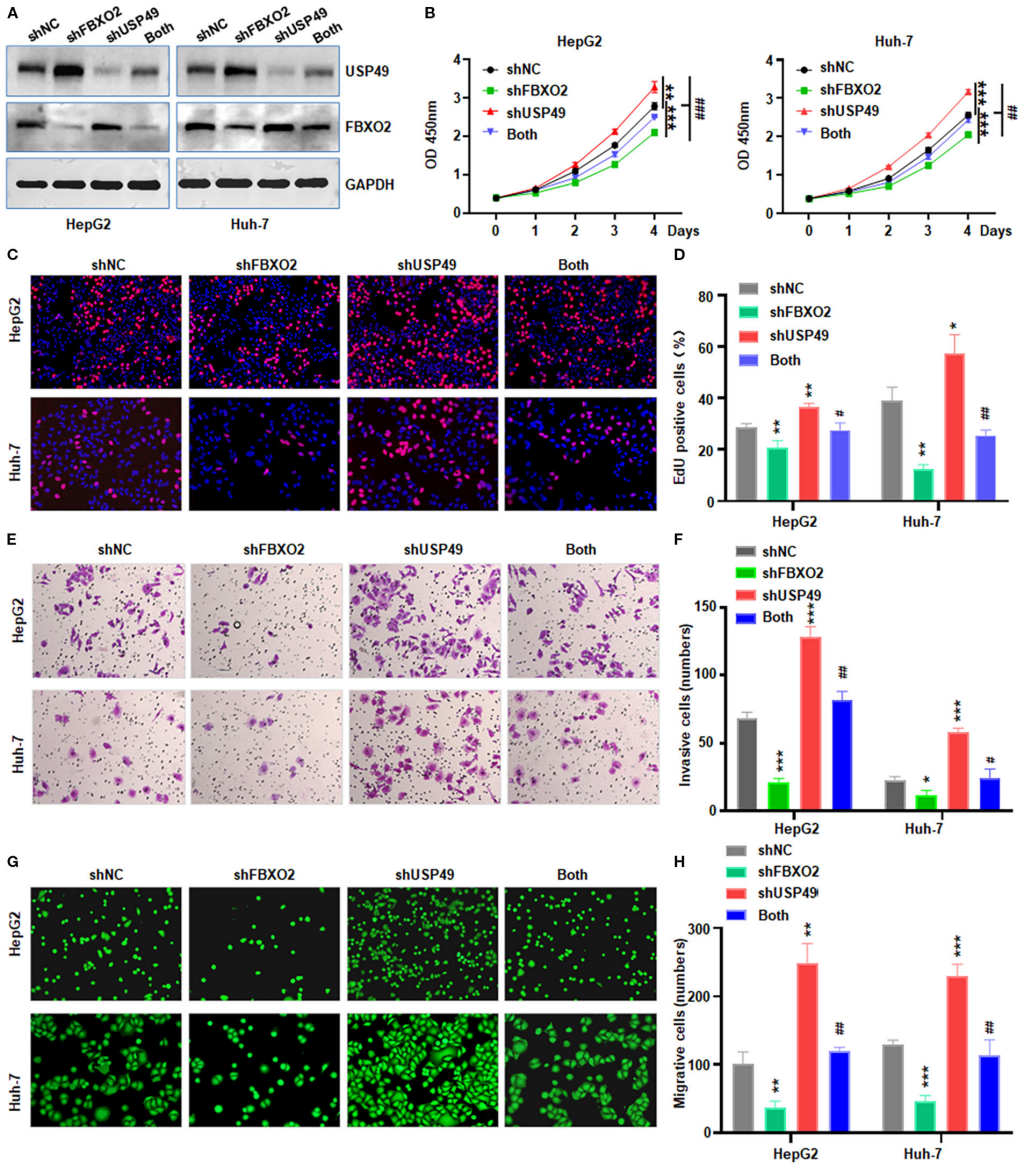
FBXO2 knockdown partially abolishes USP49-mediated proliferation, migration, and invasion in HCC cells. **(A)** Western blot analysis of FBXO2 and USP49 expression in HepG2 and Huh-7 cells infected with control, shFBXO2, shUSP49, or combined shFBXO2 + shUSP49 lentivirus. shNC: shRNA negative control; both: shFBXO2+shUSP49. **(B–D)** CCK-8 and EdU assays were performed to assess cell proliferation under the indicated knockdown conditions. **(E, F)** Transwell invasion assays were conducted to evaluate the invasive capacity of HCC cells with the indicated shRNA treatments. **(G, H)** Calcein-AM-labeled Transwell migration assays were used to assess the migratory capacity of HepG2 and Huh-7 cells under the indicated knockdown conditions. *p < 0.05, **p< 0.01, ***p < 0.001; ^#^ *p* < 0.05, ^##^ *p* < 0.01, ^###^ *p* < 0.001 vs shFBXO2 alone or shUSP49 alone.

To determine whether FBXO2 regulates HCC cell proliferation and motility through USP49, we performed co-transfection experiments in HepG2 and Huh-7 cells using shFBXO2 and shUSP49 (**Figure 6A**). CCK-8, EdU, and Transwell assays revealed that FBXO2 knockdown significantly suppressed cell proliferation, migration, and invasion. Notably, these inhibitory effects were reversed upon USP49 silencing, indicating that USP49 mediates the tumor-suppressive effects of FBXO2 depletion (**Figures 6B–H**). Together, these results demonstrate that FBXO2 promotes HCC cell proliferation and motility, at least in part, by modulating USP49 stability and activity.

### 3.7 FBXO2 deletion potentiates sorafenib sensitivity in HCC *in vitro and in vivo*

**Figure 7:**
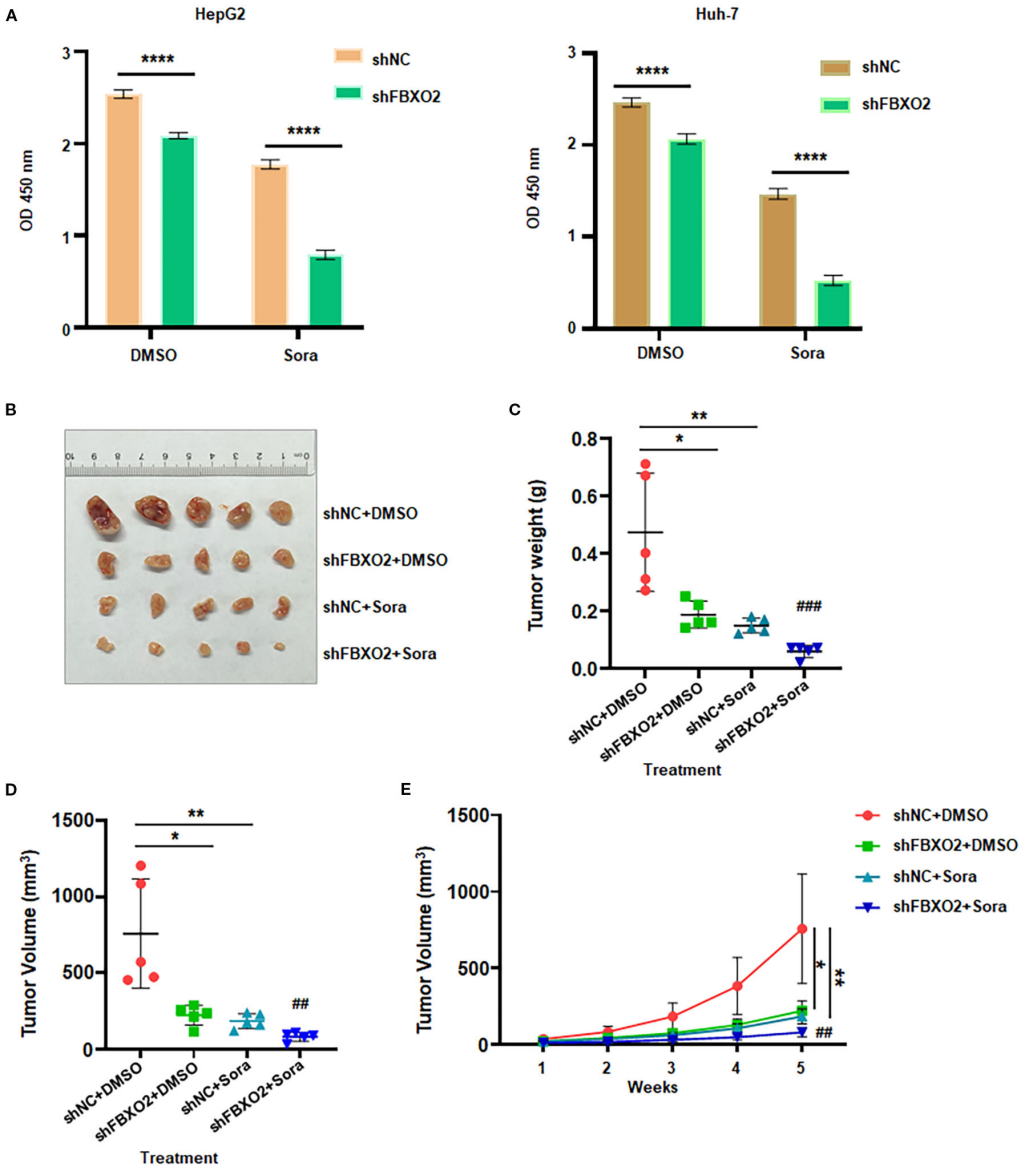
FBXO2 inhibition increases sorafenib sensitivity in human HCC. **(A)** Relative cell viability was measured in HepG2 and Huh-7 cells stably expressing shFBXO2 following 48-hour treatment with sorafenib (20 μM). **(B)** Representative images of xenograft tumors from nude mice bearing HepG2 cells transduced with shNC or shFBXO2 and treated with sorafenib or vehicle. **(C)** Tumor weights at the end of treatment. **(D)** Tumor volumes at the end of treatment. **(E)** Tumor growth curves illustrating the effects of FBXO2 knockdown and sorafenib treatment on tumor progression *in vivo*. Sora, sorafenib; *p < 0.05, **p < 0.01, ***p < 0.001; ^##^ *p* < 0.01, ^###^ *p* < 0.001 vs shFBXO2 alone or sorafenib alone.

Beyond its role in tumor progression, we investigated whether FBXO2 contributes to sorafenib resistance in HCC. Given that sorafenib is a frontline multikinase inhibitor for advanced HCC, we examined whether FBXO2 knockdown affects sorafenib sensitivity in HCC cells. FBXO2 was silenced in HepG2 and Huh-7 cells, and their response to sorafenib was evaluated (**Figure 7A**). CCK-8 assay results showed that FBXO2-depleted cells exhibited significantly increased sensitivity to sorafenib compared to control cells. Consistent with *in vitro* findings, *in vivo* experiments using HepG2 xenograft models in nude mice demonstrated that FBXO2 knockdown significantly enhanced the antitumor efficacy of sorafenib. Tumor volumes and weights were markedly reduced in the FBXO2-depleted group treated with sorafenib compared to controls (**Figures 7B–E**). Collectively, these findings suggest that targeting FBXO2 may sensitize HCC cells to sorafenib and improve therapeutic outcomes in both *in vitro* and *in vivo* settings.

## 4 Discussion

HCC is one of the leading causes of cancer-related mortality and accounts for most primary liver cancer cases worldwide (35). Despite significant advancements in cancer diagnosis and treatment, recurrence, metastasis, and drug resistance remain major clinical challenges during therapy (36). Therefore, it is essential to develop more effective therapeutic strategies aimed at reducing tumor heterogeneity and recurrence rates in HCC.

FBPs have been reported to target specific substrates and involve in tumorigenesis. FBXO32-mediated ubiquitination of Suppressor of Fused (SUFU) promotes HCC progression and lenvatinib resistance through activation of hedgehog signaling (37). F-Box and WD repeat domain containing 7 (FBXW7) regulates HCC cell growth and influences liver cell differentiation fate via targeting RNA polymerase II associated protein 2 (RPAP2) (38). FBXW7 inhibits metastasis and stemness of HCC via inducing  chromodomain helicase DNA binding protein 3 (CHD3) degradation, thereby enhancing sensitivity to oxaliplatin (39). FBXO22 targets the ribosomal protein S5 (RPS5)/AKT/hypoxia inducible factor-1α (HIF-1α)/vascular endothelial growth factor A (VEGF-A) axis, leading to enhancement of angiogenesis and metastasis in HCC (40). FBXO7 inhibits serine synthesis and tumor growth via ubiquitinating protein arginine N-methyltransferase 1 (PRMT1) in HCC (41). FBXO2 is a member of the F-box protein family and functions as a substrate recognition component within the SCF complex. It preferentially recognizes high-mannose glycoproteins and facilitates their degradation through the ubiquitin–proteasome system (UPS) (42). Due to its involvement in diverse biological processes, FBXO2 has garnered increasing research attention. It has been implicated in insulin signaling regulation by targeting the insulin receptor, positioning it as a potential therapeutic target in metabolic diseases (23). In the context of Alzheimer’s disease, FBXO2 has been associated with impaired synaptic function and cognitive decline (43). FBXO2 has become a crucial modulator of carcinogenesis, participating in a number of malignant cellular mechanisms that accelerate the development of different kinds of malignancies (44, 45). It has been known that p53 is critical in regulating cell cycle and inhibiting tumorigenesis (46). FBXO2 enhances papillary thyroid carcinoma proliferation and apoptosis by targeting p53 for ubiquitin-mediated degradation (47). By controlling the autophagy signaling system and cell cycle and functioning as an E3 ligase that ubiquitin-dependently degrades Fibrillin-1 (FBN1), FBXO2 has been shown to stimulate the growth of endometrial cancer (48). Furthermore, it has been noted that FBXO2 controls the signal transducer and activator of transcription 3 (STAT3) signaling pathway, which is essential for the growth of osteosarcoma cells (49). In this work, we provide the first proof that FBXO2 regulates USP49 expression through ubiquitination, which plays a crucial role in the development and sorafenib resistance of HCC.

Numerous investigations have demonstrated that USP49 functions as a tumor suppressor in a variety of cancer types (50, 51). *USP49* deletion significantly increased carcinogenesis in colon cancer by establishing a positive feedback loop with p53 and made HCT116 cells more resistant to etoposide-induced DNA damage (50). Furthermore, USP49 increased sensitivity to gemcitabine and inhibited proliferation in cancer cells via stabilizing FK506-binding protein 51 (FKBP51) expression, which in turn negatively mediated AKT activation (51). Similarly, USP49 inhibited cell proliferation by suppressing PI3K-AKT signaling cause lung cancer cells to enter a cell cycle halt (52). USP49 drives malignant progression of esophagogastric junction adenocarcinoma by activating the SHC binding and spindle associated 1 (SHCBP1)/β-catenin/glutathione peroxidase 4 (GPX4) signaling pathway (53). Insulin like growth factor 2 mRNA binding protein 3 (IGF2BP3)-mediated N6-methyladenosine of USP49 enhances carboplatin resistance in retinoblastoma by promoting autophagy through stabilizing Sirtuin 1 (SIRT1) (54). Cold atmospheric plasma induces USP49/histone deacetylases 3 (HDAC3)-mediated ferroptosis by enhancing lactylation-dependent p53 expression in endometrial cancer (55). USP49 promotes radioresistance in esophageal squamous cell carcinoma by stabilizing replication protein A 70 (RPA70) via homologous recombination repair (56). Additionally, it has been revealed that lncRNA hepatocellular carcinoma-associated long non-coding RNA 1 (HLNC1) binds to USP49 and destabilizes it to promote the advancement of HCC (57).

Our study demonstrates that USP49 plays a critical role in regulating HCC cell survival and motility *in vitro*. Importantly, USP49 knockdown partially rescued the inhibitory effects of FBXO2 depletion on HCC cell proliferation, migration, and invasion, further supporting its role as a downstream effector in this regulatory axis. It has been reported that FBXO2 is involved in immune response in BALB/c and C57BL/6 mice with allergic rhinitis (58). USP49 suppresses cellular antiviral responses by removing K63-linked ubiquitin chains from mediator of IRF3 activation (MITA) (59). USP49 strongly stabilizes the apolipoprotein B mRNA editing enzyme, catalytic subunit 3G (APOBEC3G) protein by deubiquitination, thereby suppressing HIV-1 replication (60). These reports indicate that FBXO2 and USP49 could participate in immune response.

## 5 Conclusions and future perspectives

In conclusion, our findings reveal that FBXO2 promotes HCC cell proliferation, invasion, migration, and resistance to sorafenib by mediating the ubiquitination and proteasomal degradation of USP49. These results identify FBXO2 as a potential therapeutic target for overcoming sorafenib resistance in HCC. Several limitations of this study should be acknowledged. A global ubiquitinome or FBXO2-interactome analysis would discover other substrates of FBXO2 in HCC. FBXO45 has been reported to target USP49 for ubiquitination and degradation, resulting in pancreatic cancer progression (27). It is unclear whether FBXO45 also targets USP49 and effects HCC progression or compensates in FBXO2-deficient cells. Since USP49 affects a range of substrates, such as Yes-associated protein 1 (YAP1), H2A histone family member X (H2AX), and SIRT1, it is required to determine which pathway is responsible for the observed phenotype in HCC. To better elucidate the role of FBXO2 in HCC, the use of liver-specific conditional FBXO2 knockout mice would be more appropriate. Additionally, the development of FBXO2 inhibitors is necessary for potential therapeutic applications, including the treatment of HCC and the reversal of sorafenib resistance. F-box proteins have been reported to regulate tumor immunity and immunotherapy (61). Further investigation is needed to determine whether FBXO2 downregulation can enhance the efficacy of immunotherapy in HCC.

## Data Availability

The original contributions presented in the study are included in the article. Further inquiries can be directed to the corresponding author.
